# ﻿*Raphiocarpus discolor* (Gesneriaceae), a new species from Guangdong, China

**DOI:** 10.3897/phytokeys.263.167423

**Published:** 2025-09-26

**Authors:** Xiao-Mao Qian, Jia-Xin Fu, Ling-Bo Ji, Bo Tang, Zi-Bing Xin, Fang Wen

**Affiliations:** 1 College of Forestry, Guizhou University, Guiyang 550025, China Guangxi Zhuang Autonomous Region and Chinese Academy of Sciences Guilin China; 2 Guangxi Key Laboratory of Plant Conservation and Restoration Ecology in Karst Terrain, Guangxi Institute of Botany, Guangxi Zhuang Autonomous Region and Chinese Academy of Sciences, Guilin, 541006, Guangxi, China Guizhou University Guiyang China; 3 Gesneriad Committee of China Wild Plant Conservation Association, National Gesneriaceae Germplasm Resources Bank of GXIB, The Gesneriad Conservation Center of China, Guilin Botanical Garden, Chinese Academy of Sciences, CN-541006 Guilin, Guangxi, China Guilin Botanical Garden, Chinese Academy of Sciences Guilin China; 4 Ecology and Environmental Science Research & Design Institute of Zhejiang Province, 109, Tianmushan Road, Hangzhou, 310007, Zhejiang, China Ecology and Environmental Science Research & Design Institute of Zhejiang Province Hangzhou China; 5 Key Laboratory of Ecological Environment Damage Control and Value Transformation of Zhejiang Province, Hangzhou, 310007, China Key Laboratory of Ecological Environment Damage Control and Value Transformation of Zhejiang Province Hangzhou China

**Keywords:** Flora of Guangdong, new taxa, plant taxonomy, *Raphiocarpus bicallosus*

## Abstract

*Raphiocarpus discolor*, a new species from Guangdong Province, China, is described and illustrated. This species is morphologically similar to *R. bicallosus* C.H.Nguyen, Aver. & F.Wen but can be distinguished by several characteristics, including the absence of prophylls, all axes bearing white pubescence, the corolla tube being externally yellow to brownish yellow with several indistinct longitudinal yellowish-brown stripes, and internally purplish red to purplish brown and glabrous. According to the IUCN Red List criteria for endangered species, this new species has been provisionally recognized as Critically Endangered (CR).

## ﻿Introduction

*Raphiocarpus* Chun (Gesneriaceae, subfam. Didymocarpoideae, tribe Tricho­sporeae) was initially described as a monotypic genus (Chun 1946). As presently understood, this genus includes about 18 species formerly placed in *Didissandra* C.B.Clarke in Candolle and Candolle (1883) from China and Vietnam (Wang et al. 1998), distributed from southern and southwestern China to northern and central Vietnam (Pham 2000; Li and Wang 2005; Phuong 2005; Phuong and Xuyen 2010; Zhang et al. 2010; Phuong et al. 2012; Chen et al. 2015; Middleton et al. 2021; GRC 2025; POWO 2025). At present, seven species are recorded in southern and southwestern China (Li and Wang 2005; Zhang et al. 2010; Phuong et al. 2012; Chen et al. 2015; Nguyen and Wen 2018; Wei 2019; Middleton et al. 2021; Wen et al. 2023).

In October 2024, during a botanical survey in Xin’an Town, Huazhou City, Maoming City, Guangdong Province, China, we collected an unknown Gesneriaceae plant with flowers and fruit. Based on its tetrandrous stamens and narrowly clavate capsules, we confirmed that it belongs to *Raphiocarpus* (Wang and Pan 1990; Li and Wang 2005). Through a comprehensive comparison of morphological characters with all documented species and type specimens of this genus, combined with a critical review of references (Wei 2010, 2019, 2023; Gao and Sun 2017; Wang 2022; Nguyen et al. 2023), we found clear differences between it and the known taxa. Therefore, we propose it as a new species, formally described as *R. discolor*, with a detailed description and illustration herein. Additionally, we provide data on its ecology, phenology, and a provisional IUCN conservation assessment.

## ﻿Materials and methods

All available specimens of *Raphiocarpus* housed in the following herbaria were examined: HITBC, IBK, IBSC, KUN, and PE. Color photographs were taken of plants in their natural habitats. Morphological observations and measurements were conducted on living plants, dried specimens, and spirit-preserved materials. The protologue descriptions of recently published *Raphiocarpus* species from Vietnam and China were carefully reviewed, and all protologues of newly published *Raphiocarpus* taxa over the past two decades were critically examined. The diagnostic characters distinguishing the new species from both previously described taxa and recently published congeners were confirmed to be unequivocally discrete. Pronounced geographic segregation was also observed among their respective distribution ranges. Technical terms were used to describe the species following Hickey and King (2000), Harris and Harris (2006), and Beentje (2016). Voucher specimens are deposited in the Herbarium of IBK.

## ﻿Taxonomic treatment

### ﻿ Raphiocarpus discolor

Taxon classificationPlantaeLamialesGesneriaceae

F.Wen, Z.B.Xin & L.B.Ji sp. nov.

F29F8EC4-2517-53BB-8FCA-D46A89509E9F

urn:lsid:ipni.org:names:77369704-1

Figs 1, 2, 3

#### Type.

China • Guangdong Province: Maoming City, Huazhou City, Xin’an Town, growing in sheltered soil beside streams under the forest on hill slopes, 21°65'N, 110°48'E, ca. 200 m, 24 October 2024, *Fang Wen & Jia-Xin Fu* (holotype: IBK00471734!; isotype: IBK00471735!).

#### Diagnosis.

*Raphiocarpus discolor* is morphologically similar to *R. bicallosus*, but it differs from the latter in its leaf blade adaxially densely white puberulent, abaxially nearly glabrous (vs. sparsely hispid adaxially and abaxially); prophylls absent (vs. present); calyx ca. 4 × 1 mm (vs. 5–6 × 2–3 mm), outside pubescent (vs. outside glandular pubescent); corolla tube outside yellow to brownish-yellow externally with several indistinct longitudinal yellowish-brown stripes (vs. tube outside yellow to orange-yellow), inside purplish-red to purplish-brown, glabrous, with two bosses inside the corolla tube placed at the base of the abaxial lobe (vs. inside yellow with more or less dense brown purple spots, hairy bosses inside the corolla tube placed at the base of the abaxial lobe); staminode ca. 6 mm long (vs. ca. 4 mm long); capsule in size 35–39 × ca. 2 mm (vs. 50–60 × ca. 3 mm) (Table 1), although there are differences between them, these distinctions remain consistently stable.

**Figure 1.**
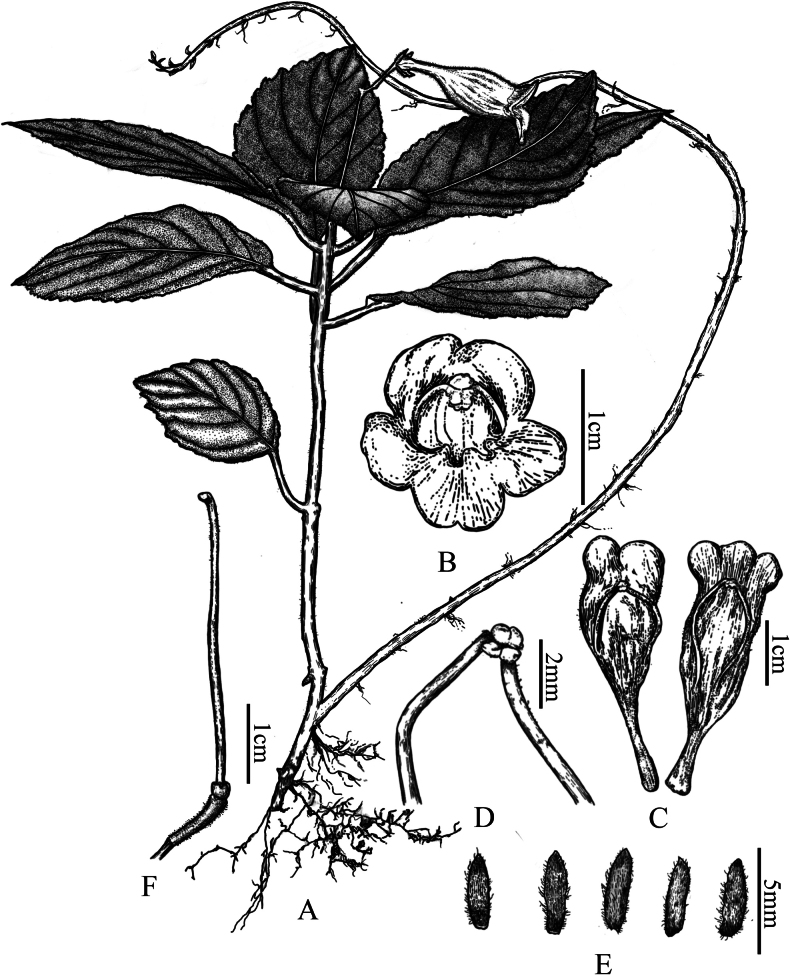
*Raphiocarpus discolor* F.Wen, Z.B.Xin & L.B.Ji. A. Habit; B. Flower, front view; C. Dissected and flattened corolla showing stamens and staminodes; D. Stamens; E. Calyx lobes; F. Pistil (drawn by Xiao-Mao Qian).

**Table 1.** The comparison of discriminative morphological characters between *Raphiocarpus discolor* and *R. bicallosus*.

Characteristics	R. discolor	R. bicallosus
Leaf blade size	3.3–10.5 × 1.7–4.9 cm, adaxially densely white puberulent, abaxially nearly glabrous	11–14 × 5–6 cm, sparsely long strigose adaxially
Prophylls	absent	present
All axes	pubescent	sparsely vertically glandular-pubescent
Cyme	1–2-flowered	3–5-flowered
Bracts	narrowly ovate to lanceolate, ca. 2 mm long, outside white pubescent	ovate to elliptic, 4–8 mm long, outside vertically glandular pubescent
Calyx	narrowly ovate to lanceolate, ca 4 mm long, ca 1 mm wide, outside vertically pubescent	broadly lanceolate to narrowly ovate lobes, 5–6 mm long, ca 2 mm wide, outside glandular-pubescent
Corolla	tube internally purplish-red to purplish-brown	tube inside yellow with more or less dense brown purple spots
Staminode	ca. 6 mm long	ca. 4 mm long
Disc	1.5–2 mm tall	1–1.3 mm tall
Capsule	linear, 3.5–4.9 cm long, ca. 2 mm in diameter	narrowly cylindrica, 5–6 cm long, ca. 3 mm in diameter

#### Description.

Perennial herb 27–50 cm tall. Stem erect or prostrate at base, slightly lignified, commonly forming extremely elongated and horizontal creeping stolons at the base of stems along the ground and rooting at the nodes or the tip, and giving rise to a new plant. Leaves opposite, often clustered at the stem tip, petiolate; petiole 1–3.7 cm long, ca. 8 mm in diameter, green to brown, densely white pubescent. Leaf blades narrowly ovate to elliptical, each pair unequal, rarely equal, with varying degrees of bilateral asymmetry, 3.3–10.1 × 1.7–5.1 cm, both surfaces green, adaxially densely white puberulent, abaxially almost glabrous, base cuneate, margin numerously dentate, apex acute to acuminate; lateral veins 5–6 on each side of midrib, adaxially inconspicuous, abaxially prominent. Inflorescence cymose, axillary or subterminal dichasium, 1–2-flowered per cyme; all axes white puberulent; peduncle 1.5–6.0 cm long, ca. 8 mm in diameter, dark green, white pubescent; bracts 2, opposite, pale green to purplish adaxially and abaxially, narrowly ovate to lanceolate, ca. 2 mm long, white pubescent, margin entire, apex acuminate; petiole 4–14 mm long, densely white pubescent. Calyx with 5-parted to nearly base, lobes, outside purplish-red to reddish-brown, inside pale green to purplish-red, narrowly ovate to lanceolate, ca. 4 mm long, ca. 1 mm wide at the widest, apex acuminate, margin entire, outside vertically pubescent, inside glabrous. Corolla tube yellow to brownish-yellow externally with several indistinct longitudinal yellowish-brown stripes, purplish-red to purplish-brown internally, limb 2-lipped, upper and lower lips externally and internally deep purplish-brown to deep purplish-black, the color of the corolla tube mouth fading downward to the midpoint of the tube, gradually deepening toward the base to purplish-black, but yellow at the 1/6 position from the base to the mouth; tube funnel-shaped, 3.2–3.7 cm long, ca. 4 mm in diameter at the base, distinctly narrowing and slightly curved from the base to the 1/3 point of the tube, with a prominent saccate swelling at the 2/3 point from the base, mouth 8–13 mm in diameter, outside vertically glandular puberulent, inside glabrous, with two bosses inside the corolla tube placed at the base of the abaxial lobe; corolla bilabiate; upper lip 2-lobed to the middle, 7–8 × 6–7 mm, lobes nearly equal, nearly semicircular; lower lip 3-lobed, lobes unequal, middle lobe 5–6 × 5–7 mm, rounded, apex emarginate, lateral lobes 4–5 × 3–6 mm, apex obtuse, slightly oblique, colored purplish-brown to violet-brown, with pinkish-purple striations along the midvein of each lobe. Stamens 4, in 2 pairs, each pair coherent at their apices, anther thecae confluent; filaments glabrous, pale yellow to purplish-red, adaxial pair ca. 1.1 cm long, adnate to the corolla tube ca. 1.5 cm above the base, abaxial pair ca. 2.2 cm long, adnate to the corolla tube ca. 2.1 cm above the base; anthers reniform constricted at the middle, ca. 1 mm long, ca. 1 mm wide, white to pale yellow, glabrous; staminode 1, clavate, ca. 6 mm long, adnate to the corolla tube ca. 2.1 cm above the base, glabrous, white to purplish-red. Disc pale yellowish, annular, obscurely 5-lobed, 1.5–2 mm tall, glabrous. Pistil 3.6–3.9 cm long; ovary linear, glabrous, 2.1–2.5 cm long, ca. 1 mm in diameter; style linear, 1.2–1.4 cm long, ca. 2 mm in diameter, sparsely glandular puberulent, ca. 1 mm in diameter; stigmas 2, equal, undivided, lobes lipped to ligulate. Capsule green when young, glabrous, 3.5–4.9 cm long, ca. 2 mm in diameter, linear, almost straight, net twisted, loculicidally dehiscing from the apex to the base when mature.

**Figure 2.**
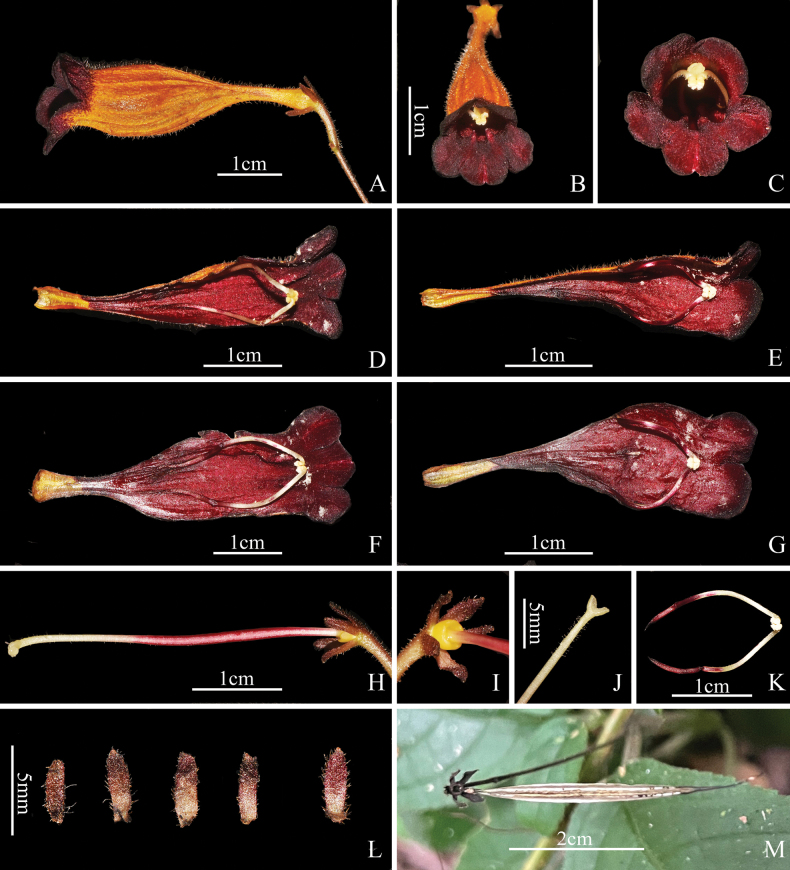
*Raphiocarpus discolor* F.Wen, Z.B.Xin & L.B.Ji. A. Lateral view of flowers; B, C. Front view of flowers; D, F. Abaxial lip; E, G. Adaxial lip; H. Pistil; I. Disc; J. Stigma; K. Stamens; L. Sepals; M. Capsule (photographed by Fang Wen & Xiao-Mao Qian).

#### Phenology.

Flowering occurs from August to October, and fruiting takes place from October to November.

#### Etymology.

The specific epithet ‘*discolor*’ is derived from the Latin ‘dis-’ (meaning ‘different’) and ‘-color’ (meaning ‘color’), referring to the color differences in different parts of the corolla.

#### Vernacular name.

Huà Zhoū Loù Doú Jù Tái (Chinese pronunciation); 化州漏斗苣苔 (Chinese name).

#### Distribution and habitat.

The new species is currently only known to be distributed in a small, gently sloping depression or valley between two hills in Xin’an Town, Huazhou City, Maoming City, Guangdong Province, China. The distribution site is a remnant of a subtropical evergreen broad-leaved forest, with the surrounding hills entirely developed into cultivation areas for eucalyptus and other economically important tree species. *Raphiocarpus discolor* typically grows on hills made of lateritic soil, at elevations of 200 meters, often influenced by a maritime climate, with additional continental climate influence in winter, and characterized by a highly pronounced monsoon climate. Accompanying species mainly include *Geophila repens* (L.) I. M. Johnst., *Gardenia* spp., *Elatostema* spp., and *Pothos* spp.

**Figure 3.**
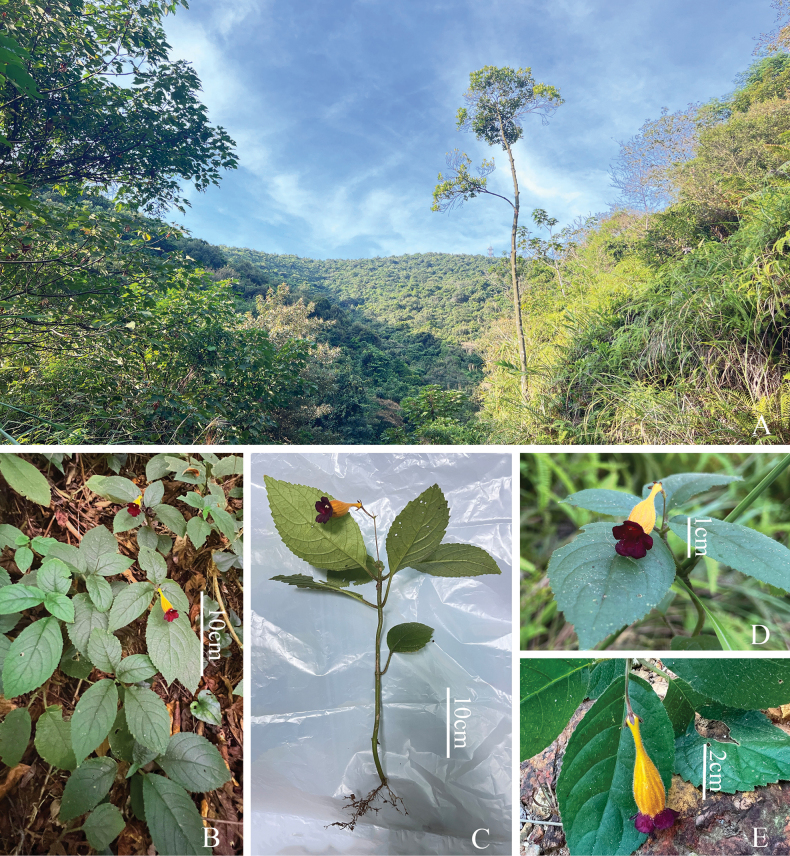
*Raphiocarpus discolor* F.Wen, Z.B.Xin & L.B.Ji. A. Habitat; B. Habit; C. Flattened flowering plant; D, E. Cymes (photographed by Fang Wen).

#### Conservation status.

Currently, only one population, consisting of approximately 100 mature individuals of *Raphiocarpus discolor*, has been discovered. The area surrounding its habitat has been entirely developed for the cultivation of economically valuable tree species, such as eucalyptus. The species *Raphiocarpus discolor* is only found in the shaded soil of a remnant woodland between two hills, not growing on rocks. Its extent of occurrence (EOO) and area of occupancy (AOO) are estimated to be less than 500 m^2^ and 10 m^2^, which is significantly smaller than the smallest EOO and AOO unit of the IUCN (2025) (100 km^2^ and 10 km^2^ for Critically Endangered under B1 and B2). Any protected area does not cover the distribution range of this species, and the growth of *R. discolor* in highly dense eucalyptus forests inhibits the development of this species. According to the IUCN Red List Categories and Criteria (IUCN 2025), the endangered level of this new species is preliminarily assessed as “Critically Endangered” [CR, B1 ab (iii, v) + B2ab (iii, v)].

#### Notes.

Compared to all known *Raphiocarpus* species, this new species exhibits distinctive characteristics, primarily reflected in its corolla tube, which is yellow to brownish yellow externally with several indistinct longitudinal yellowish-brown stripes and purplish red to purplish brown internally (whereas all known *Raphiocarpus* species have spots inside the corolla tube and diverse color variations in the corolla). Morphologically, this new species can be compared with the Vietnamese endemic species *R. bicallosus* (Nguyen et al. 2023), as they share some common features in their reproductive organs, such as the color and shape of the disc and the glabrous ovary. Additionally, the considerable geographical separation of their type localities, along with differences in climatic, hydrological, and altitudinal conditions, has likely led to distinct pollinator preferences and consequent floral color divergence. However, the differences between this new species and *R. bicallosus* are also evident, mainly in the absence of prophylls (vs. *R. bicallosus* has prophylls), all axes covered with short white pubescence (vs. *R. bicallosus* has sparsely vertically glandular-pubescent hairs on all axes), bracts covered with short white pubescence (vs. *R. bicallosus* has bracts covered with vertical glandular hairs), and the corolla tube being purplish red to purplish brown internally (vs. *R. bicallosus* has a corolla tube that is yellow internally with few or numerous brownish-purple spots). The corolla’s interior and external colors are probably transitory features, but other morphological traits allow for a clear distinction between the two taxa. The degree of transition and the existence of intermediate types in the corolla tube’s external color are difficult to identify. It is very likely that these are transitional features or that the characteristics show a continuous gradient. Transitional features are common in biological taxonomy, particularly when there is gene flow, a short interval between taxa, or when the features exhibit phenotypic plasticity under environmental effects. To improve understanding of such transitory features, statistical analysis techniques or molecular evidence may be added in the future.

## Supplementary Material

XML Treatment for Raphiocarpus discolor

## ﻿Additional information

### Use of AI

No use of AI was reported.

### Author ORCIDs

Xiao-Mao Qian https://orcid.org/0009-0004-2335-022X

Jia-Xin Fu https://orcid.org/0000-0003-0792-5383

Ling-Bo Ji https://orcid.org/0009-0009-2005-021X

Bo Tang https://orcid.org/0009-0008-4918-5313

Zi-Bing Xin https://orcid.org/0000-0002-0062-6930

Fang Wen https://orcid.org/0000-0002-3889-8835

### Data availability

All of the data that support the findings of this study are available in the main text.
